# Transforming growth factor beta-1 decreases the yield of the second meiotic division of rat pachytene spermatocytes *in vitro*

**DOI:** 10.1186/1477-7827-3-22

**Published:** 2005-06-07

**Authors:** Anne Damestoy, Marie-Hélène Perrard, Michèle Vigier, Odile Sabido, Philippe Durand

**Affiliations:** 1 INSERM U418; INRA UMR1245; Université Claude-Bernard Lyon 1, 29 rue sœur Bouvier, 69322 Lyon cedex 05, France; 2 Centre commun de Cytométrie en Flux, Faculté de Médecine, Université Jean Monnet, 42023 St Etienne, France

## Abstract

**Background:**

TGF beta and its receptors are present in both germ cells and somatic cells of the male gonad. However, knock-out strategies for studying spermatogenesis regulation by TGF beta have been disappointing since TGF beta-or TGF beta receptor-null mice do not survive longer than a few weeks.

**Methods:**

In the present study, we addressed the role of TGF beta-1 on the completion of meiosis by rat pachytene spermatocytes (PS) cocultured with Sertoli cells. Identification and counting of meiotic cells were performed by cytology and cytometry.

**Results:**

Under our culture conditions, some PS differentiated into round spermatids (RS). When TGF beta-1 was added to the culture medium, neither the number of PS or of secondary spermatocytes nor the half-life of RS was modified by the factor. By contrast, the number of RS and the amount of TP1 mRNA were lower in TGF beta-1-treated cultures than in control cultures. Very few metaphase I cells were ever observed both in control and TGF beta-1-treated wells. Higher numbers of metaphase II were present and their number was enhanced by TGF beta-1 treatment. A TGF beta-like bioactivity was detected in control culture media, the concentration of which increased with the time of culture.

**Conclusion:**

These results indicate that TGF beta-1 did not change greatly, if any, the yield of the first meiotic division but likely enhanced a bottleneck at the level of metaphase II. Taken together, our results suggest strongly that TGF beta participates in an auto/paracrine pathway of regulation of the meiotic differentiation of rat spermatocytes.

## Background

Multiplication, differentiation and survival or death of testicular germ cells are tightly regulated processes. Over the last decades it has become obvious that, in addition to the regulation exerted by the pituitary hormones (mainly FSH and LH) [1], spermatogenesis is under the control of a complex network of factors originating from both the somatic cells and the germ cells of the testis [2,3]. Moreover, it is becoming clear that hormones and intratesticular factors may compensate at least in part, for the absence of some hormones or factors, including FSH [4-6] and androgen [7-10] or luteinizing hormone [11] receptors. Thus, it is likely that synergism and/or redundancy between regulatory molecules is a characteristic of the spermatogenic process. Since most of the growth factors, cytokines and neurotrophins produced within the testis are widely expressed in the organism, the attempts to understand their role in spermatogenesis by knock-out strategies have been often disappointing. Transforming growth factor (TGFβ) is an example of such molecules. TGFβ1, TGFβ2 and TGFβ3 are expressed in the male gonad and their receptors are present in the rat testis in both somatic cells and germ cells [12-16]. TGFβ- or TGFβ receptor-null mice have been created [17-23]. However, as these mice do not survive longer than a few weeks, their usefulness for studying spermatogenesis is limited. Hence, use of culture systems associating spermatogenic cells and testicular somatic cells might be a valuable alternative to study the possible involvement of intratesticular factors such as the TGFβs on some step(s) of spermatogenesis. We [24,25] and others [26-28] have demonstrated that meiosis can proceed *in vitro *when mammalian spermatogenic cells are cocultured with Sertoli cells. In rodents, the kinetic of the meiotic process is similar *in vivo *and during the first week of culture [24,26,29] and round spermatids developed *in vitro *can produce normal offspring [30]. Such culture systems have allowed to study some cellular aspects of the meiotic process [31] or of its regulation [32]. Hence, in the present work, we addressed the role of TGFβ1 on the completion of meiosis by rat pachytene spermatocytes cultured together with Sertoli cells.

## Methods

### Isolation and co-culture of rat Sertoli cells and pachytene spermatocytes or round spermatids

Co-cultures of 21-day old rat Sertoli cells and adult rat pachytene spermatocytes (PS) or round spermatids (RS) were performed in bicameral chambers as described by Weiss *et al*. [24]. More than 90% of the cells in the PS fraction were 4C cells and more than 80% in the RS fraction were 1C cells; moreover, we found previously that 13% of elutriated PS were early PS (stages XIV-IV), 61% were middle PS (stages V-IX) and 26% were late spermatocytes (stages X-XIII) [32]. In some experiments, adult rats were injected with 5-bromo-2'-deoxyuridine (BrdU, Sigma-Aldrich, St Quentin Fallavier, France) (50 mg/kg) 14 days before they were killed, in order to label PS/diplotene spermatocytes of stages VII-XIII [32]. Under these conditions, BrdU-labelled stages XI, XII, and XIII (the only stages containing spermatocytes which may have time to differentiate into RS over a 3-day culture period [24,26,29]) represented 18% of the total number of stages containing BrdU-labelled PS/diplotene spermatocytes. These procedures were approved by the Scientific Research Agency (approval n° 69306) and conducted in accordance with the guidelines for care and use of laboratory animals.

Cells were cultured in Dulbecco's Modified Eagle's Medium (F12/DMEM) supplemented with antibiotics, NaHCO_3 _(13.4 mM), insulin (10 μg/ml), transferrin (10 μg/ml), vitamin C (10^-4^M), vitamin E (10 μg/ml), testosterone (10^-7^M), retinoic acid (3.3 × 10^-7 ^M), retinol (3.3 × 10^-7 ^M), pyruvate (1 mM) (all products from Sigma-Aldrich, St Quentin Fallavier, France), and ovine NIH FSH-20 (1 ng/ml) obtained through NIADDK at 33°C in an humidified atmosphere of 95% air : 5% CO_2_. In TGFβ1-treated wells, human recombinant TGFβ1 (10 ng/ml) (R&D Systems, Lille, France) was added in both the apical and the basal compartments of the bicameral chamber. Only basal media (with or without 10 ng/ml TGFβ1) were renewed every two days; TGFβ1 was added to the apical media at the same interval.

### Immunolabeling of cells for flow cytometry

At selected days of culture, cells were detached from culture wells by trypsinization. After counting and determination of cell viability by Trypan blue exclusion, cells were fixed with ice cold 70% ethanol.

Immunolabeling of cultured cells and flow cytometric analyses were performed as described by Vigier *et al*. [32] and Godet *et al*. [33]. After washing with phosphate-buffered saline (PBS), fixed cells were resuspended in permeabilizing buffer (0.25% Triton X-100/1% BSA/PBS) for 20 minutes on ice. The cells were then exposed to an anti-vimentin antibody (clone V9 – Dako SA, Trappes, France) at a dilution of 1:500 in blocking buffer (5% fetal calf serum/1% BSA/PBS) for 3 h at 4°C. After 3 washes in 1% BSA/PBS, the cells were incubated with fluorescein (FITC)-conjugated rabbit anti-mouse immunoglobulins (Dako SA, Trappes, France) diluted 1:60 in blocking buffer for 1 hour at 4°C. Before analysis, Hoechst 33342 (Sigma-Aldrich, St Quentin Fallavier, France) was added to the labeled cell suspension at a final concentration of 20 μg/ml.

### Flow cytometric and computer analyses

After immunolabeling, cells were analysed using a FacsStar plus Cytometer (Becton Dickinson, Le Pont de Claix, France) equipped with a 50-mW argon laser tuned to 448 nm and an INNOVA 300 ion multilined/UV laser tuned to UV. Emission of fluorescence was measured with a DF 530/30 filter for FITC, and a DF 42/44 filter for Hoechst 33342. Data acquisition and analysis were performed with CellQuest software (Becton Dickinson, Le Pont de Claix, France). Four data parameters were acquired and stored in list mode files: linear forward light scatter (FSC) and linear side angle light scatter (SSC) which roughly represent cell size and cellular granularity, respectively; logarithmic FITC (vimentin) to detect immunolabeling, and linear Hoechst to measure the DNA content of the different cell populations. Contaminating events such as debris and clumped cells were eliminated from the analysis. Each acquisition was performed on 50 000 cells negative for vimentin and allowed to identify 1C, 2C and 4C germ cells. Hence, the percentage of each category of germ cells was multiplied by the total number of cells per well in order to obtain the absolute number 1C, 2C and 4C cells.

### RNA extraction

Total RNA from cultured cells was extracted according to Chomczynski and Sacchi [34], using Trizol Reagent (Invitrogen, Cergy-Pontoise, France). The purity and the integrity of the RNA were checked spectroscopically and by gel electrophoresis respectively.

### Reverse transcription-polymerase chain reaction (RT-PCR)

Sequences corresponding to TP1 mRNA [EMBL: X07284] or p21 mRNA [EMBL: NM080782] and 16S rRNA [EMBL: XM341815] were amplified by RT-PCR as follows. Single strand cDNAs were obtained from reverse transcription (RT) of 1 μg of total RNA in a final volume of 10 μl containing 100 IU of Moloney murine leukaemia virus reverse transcriptase (MMLVRT), 1.25 mM dNTPs, 0.4 μg of oligo-dT, 10 mM DTT and 15 IU of RNase Guard (all products from Invitrogen, Cergy-Pontoise, France). RT was performed for 1 hour at 37°C. A sample in which RNA was replaced by sterile water was used as a negative control.

#### TP1/16S

coamplification reactions were carried out on 80 ng of reverse-transcribed RNA in a final volume of 30 μl containing 1,5 IU of Taq DNA Polymerase (Roche Diagnostic, Mannheim, Germany), 2 μM dNTPs, 1 μCi [^33^P]dATP (Amersham Biosciences, Orsay, France) and 12.5 pmol of each of the sequence-specific primers for TP1 and 16S (Sigma-Aldrich, St Quentin Fallavier, France) (Table 1). Amplification was performed in 5 sequential cycles (16, 18, 19, 20 and 22 cycles) at 94°C for 1 minute, 58°C for 1 minute and 72°C for 1 minute.

**Table 1:** Sequences of the primers used to amplify mRNAs for TP1, p21 and 16S RNA

**RNA**	**Primers**	**Product size**(nt)
**TP1 **[EMBL: X07284]*	5'_26 _– CCAGCCGCAAACTAAAGACTCATGG – 3'	175
	5'_200 _– AGCTCATTGCCGCATTACAAGTGGG – 3'	

**p21 **[EMBL: NM080782]*	5'_107 _– GACCTGTTCCACACAGGAGCAAAGT – 3'	145
	5'_251 _– GTCTCAGTGGCGAAGTCAAAGTTCC – 3'	

**16S **[EMBL: XM341815]*	5'_138 _– TGGGCTCATCAAGGTGAATGG – 3'	268
	5'_405 _– GGTCCGATCGTACTGGATGAGGATA – 3'	

nt : nucleotides

* gene bank accession numbers.

#### p21/16S

coamplification reactions were carried out on 80 ng of reverse-transcribed RNA in a final volume of 30 μl containing 1,5 IU of Taq DNA Polymerase (Roche Diagnostics), 3 μM dNTPs, 1.5 μCi [^33^P]dATP (Amersham Biosciences) and 12.5 pmol of the sequence-specific primers for p21 (Sigma-Aldrich) (Table 1). In a first step, amplification was performed for 6 cycles at 94°C for 1 minute, 58°C for 1 minute and 72°C for 1 minute. Then, 12.5 pmol of the sequence-specific primers for 16S were added and amplification was continued and performed in 5 sequential cycles (20, 22, 24, 26 28 cycles).

The amplified products were electrophoresed in parallel with size markers on 3% Metaphor Agarose gels (Tebu, Le Perray-en-Yvelines, France). The bands were cut from the gels and melted in distilled water at 100°C. Radioactivity was measured using a liquid scintillation counter.

### TGFβ bioassay

TGFβ concentrations in apical culture media were determined using mink lung epithelial cell (CCl-64), according to Danielpour *et al*. [35]. Briefly, CCl-64 cells were maintained in 75 cm^2 ^flasks in F-12 DMEM medium supplemented with 10% foetal calf serum. In 0.32-cm^2 ^wells (96-well plate), aliquots (5–10 μl) of conditioned medium heated (or not) at 80°C for 5 minutes were added in 100 μl assay medium supplemented with 5% foetal calf serum. After 1 hour at 37°C, CCl-64 cells in logarithmic growth phase were trypsinised, washed once with assay medium, and plated at 3.10^4 ^cells/100 μl in wells. CCl-64 cells were cultured overnight, followed by pulse labelling with [^3^H]thymidine (40–60 Ci/mmol; Amersham Biosciences, Orsay, France) at a final concentration of 1 μCi/ml for 4 hours at 37°C. The cells were fixed three times with 250 μl methanol-acetic acid (3:1, vol/vol) and were washed twice with 80% methanol. ^3^H-labeled DNA was extracted by 250 μl 0.4% deoxycholate 0.5 N NaOH. Radioactivity was measured by a liquid scintillation counter. In some cases TGFβ bioactivity of culture media was determined in the presence of a TGFβ1/2/3 blocking antibody (clone 1D11, R&D Systems, Lille, France).

### Immunocytochemical studies on cultured cells

At selected days of culture, cells were fixed directly in the well with Bouin's fixative for 20 minutes at room temperature.

#### 1-Immunocytochemical reaction against phosphorylated Histone H3 (pH3)

After washing with PBS, fixed cells were permeabilized with 0.03% TritonX-100 in PBS for 30 minutes. The immunocytochemical reaction was performed with the Dako LSAB kit (Dako SA, Trappes, France) as follows. Cells were incubated with 3% H_2_O_2 _for 5 minutes, rinsed with PBS, then incubated with a monoclonal mouse anti-pH3 antibody (Upstate Biotechnology, Euromedex, Mundolsheim, France) at 1:200 dilution in Dako's antibody diluent for 3 hours at room temperature. After three washes with PBS, the staining reaction was performed using the avidin-biotin-peroxydase complex and amino-3-ethyl-9-carbazole (AEC) as a chromogen.

#### 2-BrdU immunochemical reaction

The same protocol as described above was performed, but denaturation of DNA was required before the immunoreaction. Cells were incubated for 5 minutes with NaOH 0,07N diluted in alcohol:water (v:v) and then dipped in PBS for 10 minutes. Cells were incubated with a monoclonal mouse anti-bromodeoxyuridine antibody (Dako SA, Trappes, France) diluted 1:100 overnight at 4°C. After three washes with PBS, the staining reaction was performed using avidin-biotin-peroxidase complex and AEC as a chromogen.

The membrane of the insert was cut off and mounted in Dako aqueous mounting medium.

The morphologic identification and counting of the different types of germ cells was performed as previously described [32].

### Statistical analysis

ANOVA was used to compare between data from more than two groups. Paired Student's t test was used to assess statistical differences between control and TGFβ1-treated cells.

## Results

### Effects of TGFβ1 on the number and viability of total germ cells and somatic cells

When about 3.10^5 ^purified PS were cocultured with about 3.10^5 ^Sertoli cells, both the number of total cells (somatic cells plus germ cells) and their viability decreased slightly during the culture period, as expected [24]. For both parameters, the decreases were similar in control and TGFβ1-treated cultures. On day 7, the percentages of cells, compared with day 1, were 79 ± 8 and 79 ± 10% (m ± SEM, n = 7) for controls and treated cells; the viabilities were 71 ± 4 and 69 ± 3%, respectively. Every day they were studied, the percentages of germinal cells and of somatic cells were similar in control and TGFβ1-treated cultures but the percentage of germ cells decreased during the culture, whereas that of somatic cells increased 1.3 fold (data not shown). This indicates that germinal cells were lost at a similar rate irrespective the culture conditions, whereas the number of somatic cells remained constant during the culture period.

### Effects of TGFβ1 on the number of germ cells of different ploidy and on the amount of TP1 mRNA

**Figure 1:**
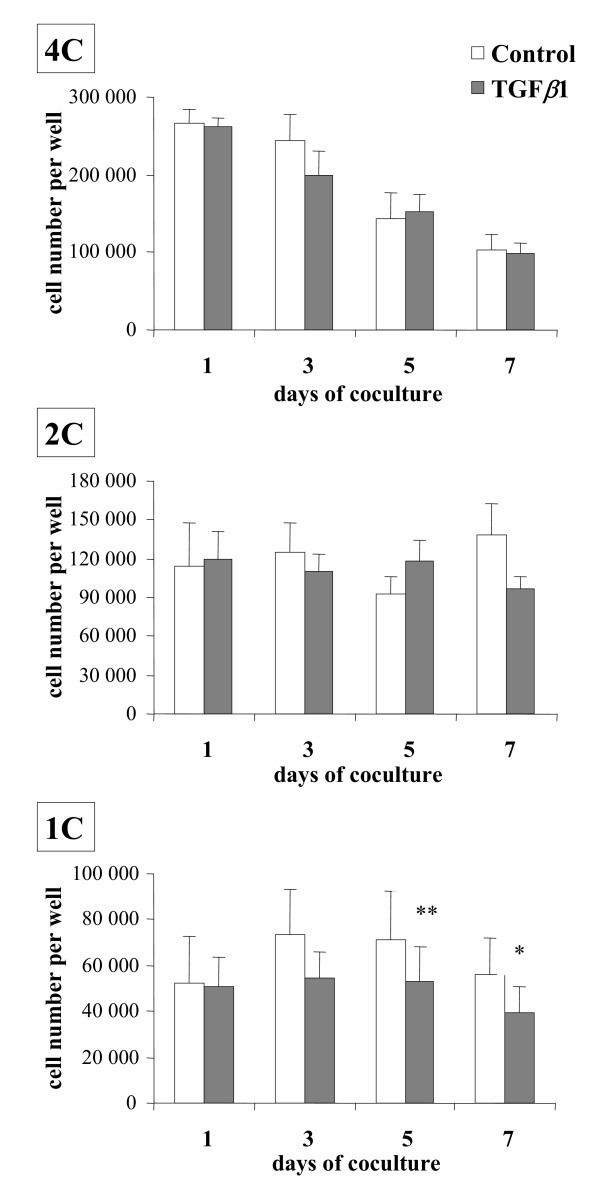
Changes in the number of germ cells of different ploidy in cocultures of Sertoli cells and PS (4C cells) maintained in the absence (control) or presence of 10 ng/ml TGFβ1 (TGFβ1). Results are the mean ± SEM of seven experiments. ** p < 0.01 vs control; * p < 0.05 vs control.

**Figure 2:**
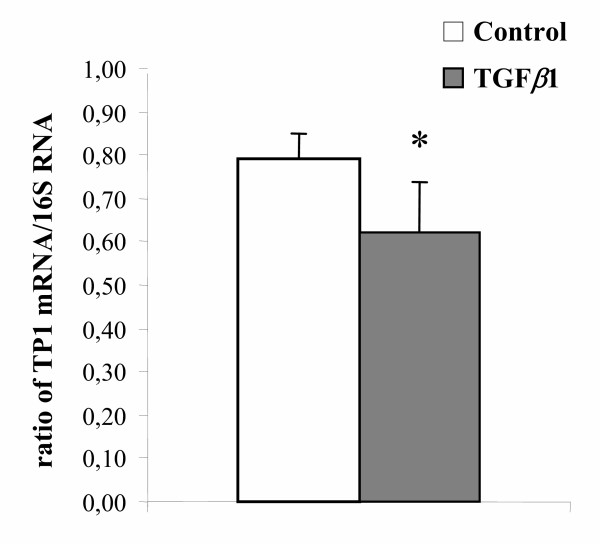
Effect of TGFβ1 on the TP1 mRNA/16S rRNA ratio on day 14 of cocultures of Sertoli cells and PS. PS were cocultured with Sertoli cells for 14 days either in the absence (control) or presence of 10 ng/ml TGFβ1. On day 14 of coculture, total RNA was extracted and TP1 mRNA and 16S rRNA were coamplified by RT-PCR, as described in the Materials and Methods section, and their ratio were calculated. Each point is the mean ± SEM of four experiments. * p < 0.05 vs control.

The number of seeded PS (4C cells) decreased 2.5 fold between day 1 and day 7 in control and TGFβ1-treated cultures (both p < 0.01) and no difference was observed between these two conditions on any studied day (Fig. 1). Under our culture conditions, some of the PS differentiated into RS (1C cells) [24,31,32]. However, when TGFβ1 was added to the culture medium, the number of *in vitro *formed RS was significantly lower on days 5 and 7 than in control cultures (p < 0.01 and p < 0.05 respectively). By contrast, no significant difference was ever observed in the population composed of secondary spermatocytes and of doublets of RS (2C cells) (Fig. 1). In addition, the amount of TP1 mRNAs (specific to the haploid state), assessed by the TP1/16S ratio on day 14, of TGFβ1-treated cultures was lower than that of controls (p < 0.05) (Fig. 2).

### Effects of TGFβ1 on the survival of RS cultured with Sertoli cells

Taken together, these results suggest that TGFβ1 inhibited the differentiation of PS into RS and/or affected the survival of RS. Therefore, in the next series of experiments, purified RS were cocultured with Sertoli cells for 5 days either in the absence or presence of TGFβ1 and the number of RS was determined daily between days 2 and 5. As expected [24,32], there was a steady decrease in the number of RS during the culture period. The regression line fitting the number of remaining RS on each day allowed the calculation of the half-life of RS which was identical in the absence or presence of TGFβ1 (Table 2).

**Table 2:** Half-lives of RS cocultured with Sertoli cells in the absence or presence of TGFβ1

**Treatment**	**Half-life **(days)
Control	1.9 ± 0.2
TGFβ1	1.9 ± 0.2

Elutriated RS were cocultured with Sertoli cells for 5 days in the absence (control) or presence of 10 ng/ml TGFβ1 and the number of remaining RS was assessed by flow cytometry daily between day 2 and day 5 allowing the determination of their half-life in culture. Each value is the mean ± SEM of three different experiments.

### Effects of TGFβ1 on the number of BrdU-labeled meiotic germ cells and the number of meiotic metaphases

Hence, the above results indicate that most likely, TGFβ1 was able to inhibit, at least partly, some step(s) in the differentiating pathway of PS into RS. In an attempt to identify this step, in the next experiments, BrdU-labeled PS/diplotene spermatocytes of stages VII-XIII were cocultured with Sertoli cells in the absence or presence of TGFβ1 ; then, the number of BrdU-labeled PS, BrdU-labeled secondary spermatocytes (SII) and that of BrdU-labeled RS were assessed by microscopic examination. The data presented in Table 3 show that TGFβ1 treatment modified neither the number of PS nor the number of SII on any studied day, but decreased the number of RS on day 3 of culture. In addition, the number of metaphases seemed to increase a little on day 3 in the presence of TGFβ1.

**Table 3:** Numbers of BrdU-labeled PS, of BrdU-labeled secondary spermatocytes (SII) and BrdU-labeled RS on day 1 and day 3 of coculture of BrdU-labeled PS/diplotene spermatocytes of stages VII-XIII and Sertoli cells in the absence or presence of TGFβ1

	Number of PS/mm			Number of SII/mm			Number of RS/mm			Number of metaphases		
	
Days of culture	-		+	-		+	-		+	-		+
1 (n = 3)	67.6 ± 14.5	NS	63.9 ± 17.4	1.3 ± 0.3	NS	1.7 ± 0.3	0.1 ± 0.1	NS	0.1 ± 0.1	0	NS	0
3 (n = 7)	42.7 ± 5.1	NS	33.4 ± 6.1	4.1 ± 0.8	NS	3.8 ± 0. 9	5.7 ± 1.3	p < 0.05	4.2 ± 1.3	1.3 ± 0.2	NS	1.8 ± 0.3

BrdU-labeled PS/diplotene spermatocytes were cocultured for 3 days in the absence (-) or presence (+) of 10 ng/ml TGFβ1. On days 1 and 3, the numbers of BrdU-labeled PS, BrdU-labeled SII, BrdU-labeled RS and BrdU-labeled metaphases were determined by microscopic examination of the culture wells and expressed as the number of each cell type per mm of well and on the width of the microscope field (objective × 100). About 500 BrdU-labeled cells were counted in each condition and in each experiment.

Each value is the mean ± SEM ; n : number of experiments.

NS : not significant.

**Figure 3:**
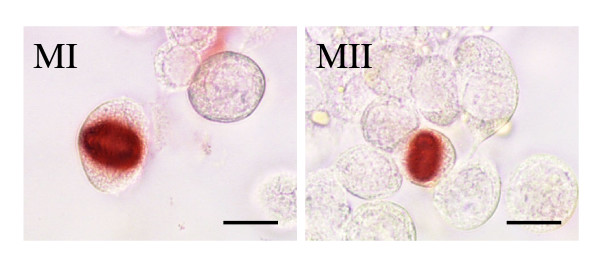
Staining of metaphase I (left) and metaphase II (right) by a Ser 10 phosphorylated histone H_3 _antibody in cocultures of PS and Sertoli cells (day one of coculture; bars represent 10 μm).

Therefore in the next experiments, cultured cells were stained with a Ser 10 phosphorylated histone H3 antibody in order to label cells in the division phase [36,37] and the numbers of metaphase I and metaphase II were recorded at the level of the whole germ cell population, not only BrdU-labelled cells (Fig. 3 and Table 4). Very few meiotic metaphase I cells were ever observed both in control and in TGFβ1-treated cultures precluding meaningful statistical analysis. By contrast, higher numbers of metaphases II were present on any studied day. Furthermore, the number of metaphase II was significantly enhanced over control by TGFβ1 on days 3 and 5 of the experiment (p < 001 and p < 0.05 respectively).

**Table 4:** Numbers of metaphase I and metaphase II on different days of coculture of PS and Sertoli cellsin the absence or presence of TGFβ1

	**Number of metaphases I/mm**			**Number of metaphases II/mm**		
**Days of coculture**	**Control**		**TGFβ1**	**Control**		**TGFβ1**

1	0.09 ± 0.02	NT	0.03 ± 0.01	1.8 ± 0.3	NS	1.8 ± 0.3
3	0.15 ± 0.04	NT	0.05 ± 0.03	2.3 ± 0.3	p < 0.01	3.1 ± 0.4
5	0.04 ± 0.02	NT	0.03 ± 0.01	0.7 ± 0.1	p < 0.05	0.9 ± 0.2

PS were cocultured for 5 days in the absence (control) or presence of 10 ng/ml TGFβ1. On days 1, 3 and 5, the numbers of metaphase I and of metaphase II were determined by microscopic examination of the culture wells and expressed as the number of each type per mm of well and on the width of the microscope field (objective × 100). 400 to 1700 metaphase II were counted in each condition, whereas the number of metaphase I which could be counted ranged between 15 and 75 precluding meaningful statistical analysis.

Each value is the mean ± SEM of 5 different experiments.

NS : not significant ; NT : not tested.

### Effects of TGFβ1 on the expression of p21 mRNA

In an attempt to explore the mechanism of this inhibitory effect of TGFβ1 on the cell cycle of the meiotic cells, in the following experiments the amount of mRNA encoding for the cyclin-dependent kinase inhibitor p21^waf1/cip1 ^was assessed on days 1, 2, and 3 of cocultures performed in the absence or presence of TGFβ1. The p21/16S ratio was similar in control and TGFβ1-treated cultures on any tested day (data not shown).

### Changes in the concentration of TGFβ bioactivity during coculture of PS with Sertoli cells

Secretion of TGFβ1 by Sertoli cell-germ cell cocultures has been demonstrated by western blotting [38]. Therefore in the following experiments we studied whether a TGFβ-like bioactivity was released under our culture conditions. No TGFβ-like bioactivity could be detected in the media of the apical compartments of the culture chambers prior heat-treatment (data not shown). By contrast significant amounts were observed following heat activation; the concentration increased almost linearly with the time of culture from 0.8 ± 0.1 on day 3, up to 4.0 ± 0.2 ng/ml on day 15 (m ± SEM, n = 3). This bioactivity was completely blocked by a TGFβ neutralizing antibody directed against TGFβ1,2,3 (data not shown).

## Discussion

To our knowledge, there has been no previous study on the effect of TGFβ on the meiotic differentiation of male germ cells. Yet it has been shown that germ cells synthesize TGFβ and are targets for the peptide (see [39] for review). However, TGFβ-null mice die in pre-or neonatal stages, making it virtually impossible to investigate the significance of TGFβ in the late stages of spermatogenesis [17-23]; hence we have studied the effect of TGFβ on meiotic differentiation of spermatogenic cells by using a system of culture of rat PS previously established in our laboratory [24,31,32]. No effect of TGFβ1 was observed on the number of remaining PS or Sertoli cells on any day under our culture conditions. Likewise TGFβ1 did not modify the half-life of seeded RS. Hence, it seems reasonable to conclude that the lower number of RS observed in TGFβ1-treated wells was due to an inhibitory effect of this factor on the transformation of PS into RS. This assumption appears substantiated by the lower content in TP1 mRNAs (specific to the haploid state) of TGFβ1-treated cultures of 14 days. Screening of TP1 mRNA content was performed on day 14 for two reasons : i) the total number of RS in TGFβ1-treated cultures was significantly lower than in controls only from day 5 onward (Fig. 1). ii) We showed previously that expression of TP1 appears to be important only from steps 5–6 of spermiogenesis, that is, 4–5 days after completion of meiosis [40]. However, the possibility that TGFβ1 might decrease the transcription of TP1 and/or the half-life of its mRNA cannot be excluded.

An effect of TGFβ1 was observed only beyond one day of culture, which is the time necessary to the more advanced elutriated spermatocytes (stage XIII) to differentiate into secondary spermatocytes under our culture conditions [31], and affected the 1C-cell population but not the 2C-cell population. Moreover, cytological monitoring of the fate of BrdU-labeled PS showed that the numbers of SII were similar in the absence or presence of TGFβ1, whereas the number of BrdU-labeled metaphase seemed enhanced by this factor. This latter point was confirmed, at the level of the whole germ cell population, by use of a Ser 10 phosphorylated histone H3 antibody which allowed us to show that the number of second metaphases was enhanced in the presence of TGFβ1. Taken together, these results suggest that TGFβ1 did not change greatly, if any, the yield of the first meiotic division, but likely enhanced a bottleneck resulting in an increase in the number of metaphase II.

Progression of cells through the cell cycle requires assembly and activation of cyclin-CDK complexes [41]. CDK activity can be regulated by changes in cyclin or CDK levels, by post-translational modifications of the CDK subunit and by formation of a complex with a set of proteins called CKI [42]. P21^waf1/cip1 ^belongs to the CKI family and binds to CDK2-cyclin A/E complexes [43] which are active during the G1 phase, S phase and/or the G2/M transition. Moreover, CDK2 and cyclin A1 appear essential for meiosis [44-46]. Several studies have shown that TGFβ1 induces p21^waf1/cip1 ^expression [47,48]. In the testis, Beumer *et al*. [49] have suggested that p21^waf1/cip1 ^could be important during the meiotic phase of spermatocytes as it is present in PS of stage V up to step 5 RS. As the work stands, we did not observe an increase in the amount of p21^waf1/cip1 ^mRNA in TGFβ1-treated cultures. However, additional mechanisms appear able to mediate the cell cycle arrest caused by TGFβ [43,50-52].

*In vivo*, TGFβ1 is present mainly in spermatocytes and RS [13,53] and rat testicular germ cells and Sertoli cells release different types of bioactive TGFβs *in vitro *( [12,38,53] and present results).

Immunohistochemistry studies have localized both type I (TβRI) and type II (TβRII) transducing receptors for TGFβ to the seminiferous epithelium in the rat testis [12,15,16]. Moreover, Smad2 and Smad3 that mediate intracellular signaling of the TGFβ superfamily from the cell surface to the nucleus are present in meiotic cells [54,55] and TGFβ can regulate gene expression in these cells [56]. The amounts of TGFβ-like bioactivity measured in our culture media fit quite well with those reported in cell culture supernatants of different testicular cell types by Haagmans *et al*. [53]. Moreover, these concentrations are quite in the range of the TGFβ concentrations required to regulate Cyp19 gene expression in isolated rat PS and RS [56] or to perturb the inter-Sertoli tight junction permeability barrier *in vitro *[57]. Furthermore, the work of Haagmans *et al*. [53] indicates that latent TGFβ released by primary cultures of germ cells and Sertoli cells can be converted into a bioactive form in the culture medium. Taken together, these results strongly suggest that TGFβ can be involved in an auto/paracrine pathway of regulation of the meiotic differentiation of rat spermatocytes. In experiments not reported here, we tried to inhibit the meiotic action of "endogenous" TGFβ with the anti-TGFβ antibody we used in the above bioassay ; however, the acid wash required to remove the antibody before counting the germ cells by either method precluded accurate analyses.

It might be argued that the effects of TGFβ1 observed in the present study are relatively minor. However, it must be underlined that their amplitude is quite similar to the effects of TGFβ on [^3^H]thymidine incorporation by rat seminiferous tubule segments [58] or on the reduction of the number of rat gonocytes [59]. Moreover, it must be emphasized that the rather high standard errors of means, both in control and TGFβ1-treated cultures, were much more due to variations between the different experiments, as occurs often *in *vitro, than to variations of the effect of TGFβ1 treatments. Indeed, for instance, of the seven experiments reported in Fig. 1 or in Table 3 (day 3), six or seven exhibited a lower number of RS in TGFβ1-treated wells vs controls. Therefore it is not surprising that statistical analysis resulted in significant differences. In addition, it is clear that synergism and/or redundancy between local regulatory factors are a characteristic of the spermatogenic process ([2,32] and unpublished results). Hence it is not unexpected that many regulatory molecules, produced within the testis, may have somewhat limited effect when tested in the presence of the other factors regulating, in the same sense, the same step of spermatogenesis. As for the effects of TGFβ1 observed in the present work, two points should be emphasized: i)it is most likely that the amplitude of the effects of exogenous (added) TGFβ1 were limited by the TGFβ produced by our germ cell-Sertoli cell cocultures (see above); ii) in other studies not reported here, we observed that NGF, which is produced by PS and RS both *in vivo * [60, 61] and under our culture conditions (M.H. Perrard, unpublished results) is also able to negatively regulate the meiotic divisions of rat PS. Thus, it is very likely, that the presence of endogenous NGF also limited the effects of TGFβ1 observed in the present studies. Indeed, such a synergism/redundancy appears to be a major problem when exploring the local regulations of spermatogenesis which makes the knowledge of this topic far from being complete. Additional studies are now required to understand the mechanism of this action of TGFβ1 and why it is likely to be on the second meiotic division.

## Conclusion

These *in vitro *results, together with previous studies showing the presence of TGFβ and its receptors in both the germ cells and the somatic cells of the male gonad, suggest strongly that TGFβ1 participates in an auto/paracrine pathway of regulation of the meiotic differentiation of rat spermatocytes.

## Authors' contributions

AD participated in the design of the study, performed cultures and immunocytochemical and flow cytometry analyses and carried out PCR experiments. MHP participated in the design of the study, in the cultures and performed immunocytochemical studies. MV participated in the design of the study, in the cultures and in the PCR experiments, and performed TGFβ bioassays. OS carried out flow cytometry analyses. PD designed and coordinated the experiments, participated in the cultures, performed statistical analyses and drafted the manuscript. All authors read and approved the manuscript.
